# Evaluation of the RIFER procedure in treating high intersphincteric anal fistulas

**DOI:** 10.3389/fmed.2025.1589278

**Published:** 2025-05-16

**Authors:** Yan Ding, Yahong Xue, Yaqiu Miao, Huiting Zhu, Rui Ye, Xiaofeng Wang

**Affiliations:** ^1^Colorectal Surgery Center, Nanjing Hospital of Chinese Medicine Affiliated to Nanjing University of Chinese Medicine, Nanjing, China; ^2^Graduate College, Nanjing University of Chinese Medicine, Nanjing, China

**Keywords:** RIFER, high intersphincteric anal fistula, functional outcome, complications, retrospective study

## Abstract

**Introduction:**

A high intersphincteric anal fistula is a common anorectal disease that is challenging to treat due to high recurrence rates and has the risk of sphincter damage, which can lead to incontinence. This study aimed to evaluate the efficacy and safety of the rectal incision, fistula excision, and reconstruction (RIFER) procedure for treating high intersphincteric anal fistulas.

**Methods:**

Twenty-six patients with high intersphincteric anal fistulas who were admitted to Nanjing Hospital of Traditional Chinese Medicine between September 2021 and March 2024 and underwent the RIFER procedure were included. Patients were followed up for 6 months, and treatment efficacy, recurrence, and postoperative complications after the RIFER procedure were assessed.

**Results:**

The surgical cure rate of patients treated with RIFER was 100%, with no recurrence or postoperative complications during the follow-up period. The average wound-healing time was 45.40 days. Functional scoring indicators, such as the visual analog and Wexner anal function scores, improved at different postoperative time points. After the RIFER procedure, the incision scar score of most patients (18 of 25) was 0, and none of the patients reported keyhole-like anal deformities. The mean hospital stay was 10.15 days.

**Conclusion:**

The RIFER procedure demonstrated remarkable efficacy and safety in the treatment of high intersphincteric anal fistulas, with no recurrence or postoperative complications. This procedure is suitable for treating high intersphincteric anal fistulas in clinical practice.

## Introduction

High intersphincteric anal fistulas are located between the internal and external anal sphincters at a high position and are often accompanied by multiple branches and fistulous openings, making surgical treatment challenging. The etiology of anal fistulas remains unclear and is generally believed to be related to diseases, such as perianal abscesses, anal fissures, and anorectal abscesses (1). Long-term diarrhea, constipation, Crohn’s disease, immunosuppression, and a history of perianal trauma can also increase the risk of developing anal fistulas (2–5). The global incidence of anal fistulas ranges from 8 to 25%. In China, this proportion ranges from 1.67 to 3.6%. Moreover, this disease is more common among young people aged 20 to 40 (6–8). Owing to their location and complex pathological structure, high-level anal fistulas are the most difficult to treat and one of the most challenging diseases in anorectal surgery, with a recurrence rate of approximately 10% (9). Owing to its location, it often leads to repeated perianal infections, swelling, pain, and scar formation (10). In severe cases, it may even cause serious complications, such as multiple complex anal and rectovaginal fistulas, which greatly affect the quality of life of patients and threaten their safety (11).

Surgery is the primary treatment modality for high anal fistulas (12, 13) and is based on three key principles: identifying the anal canal and its internal opening, excising the fistula tract, and preserving the function of the anal sphincter (14). High anal fistulas can extend to the deep part of the external anal sphincter or even further. Fistular tracts are usually complex, making it difficult to accurately identify the course of the fistula and its relationship with the sphincter during surgery, resulting in a relatively low cure rate (15). The effectiveness of anal fistula surgery is not solely determined by the immediate cure rate. Current research focuses on colorectal and anal surgery to effectively reduce postoperative complications, maintain normal function of the anal sphincter, alleviate postoperative pain, and improve quality of life (16).

Numerous studies have explored various treatment modalities for high intersphincteric fistulas (17–20). Fistulectomy, a common surgical approach, involves complete excision of the fistula tract, branches, and abscess cavity. As reported by Hirschburger et al., this method aims to eliminate all the potential sources of infection. However, they reported that the fistulas healed but recurred within the observation period in 5 patients (10%). In one patient (2%), the fistula did not heal. Mild fecal gas incontinence occurred in three patients, and one patient with grade 2 fecal incontinence showed improvement (17). The seton technique, a traditional Chinese medical approach, has also been widely used. There are two main types: the cutting-seton and the loose-seton. The seton technique is a traditional Chinese medical treatment that uses the constrictive force of silk threads or rubber bands to slowly cut through the muscles encircled by the fistula tract, thereby preserving the anatomical and physiological functions of the anus. Studies have shown that the cutting-seton technique can be used as a radical treatment method for high anal fistulas, with relatively low incontinence and recurrence rates ranging from 8 to 22%. However, this technique often leads to complications such as incontinence and severe postoperative pain (18, 19). In contrast, the loose seton technique can improve drainage and accelerate healing with a low risk of sphincter damage (20).

Although various treatment methods have been applied to high-level perianal fistulas, their efficacy remains controversial. Despite attempts to completely remove the diseased tissue, fistulectomy frequently leads to severe postoperative pain, which can greatly affect the patient’s quality of life during the recovery period. Moreover, owing to the complex anatomical location of high intersphincteric fistulas, accurate excision without damaging the anal sphincter is challenging. This often results in sphincter damage, leading to incontinence in some patients. Regarding the seton technique, the cutting seton, although it can gradually cut through the muscles encircled by the fistula tract to preserve anal function, causes significant postoperative pain and has a relatively high risk of incontinence. The loose seton, while it reduces the risk of sphincter damage, may not be sufficient to completely eliminate complex fistulas, especially those with multiple branches and deep-seated abscesses. These limitations highlight the need for more effective treatment approaches. The rectal incision, fistula excision, and reconstruction (RIFER) procedure consists of three key steps: first, a rectal incision is made to fully expose the surgical field and facilitate subsequent operations; second, the diseased fistula tissue is completely excised to eliminate sources of infection and other lesions; and finally, the rectum and surrounding tissues are reconstructed to restore their normal physiological state, treat the disease, reduce complications, and improve the quality of life of patients. However, research on the efficacy of RIFER is lacking. Therefore, we conducted this observational study to evaluate the efficacy and safety of RIFER for the treatment of high intersphincteric anal fistulas.

## Materials and methods

### Study setting

This retrospective study included patients with high intersphincteric anal fistulas who underwent the RIFER procedure at the Nanjing Hospital of Traditional Chinese Medicine in China between September 2021 and March 2024. The inclusion criteria were as follows: (1) diagnosis of a high intersphincteric anal fistula, regardless of the presence or absence of branches, by preoperative magnetic resonance imaging (MRI) and/or endoluminal B-ultrasound examination. A high intersphincteric anal fistula refers to an anal fistula in which the fistula tract is located between the deep part of the external anal sphincter and the puborectalis muscle; (2) age between 18 and 65 years; and (3) quiescent fistula tract with no obvious signs of infection. Quiescent fistulas were selected to ensure a more homogeneous study population and to minimize the potential confounding factors associated with active inflammation. Active inflammation in non-quiescent fistulas can lead to increased tissue edema and bleeding during surgery and may affect the accuracy of surgical procedures and the assessment of surgical outcomes. The exclusion criteria were as follows: (1) specific anal fistulas caused by Crohn’s disease, ulcerative colitis, etc.; (2) anal fistulas caused by trauma; (3) presence of severe anorectal diseases, such as combined digestive tract tumors; (4) presence of diseases that may affect wound healing, such as diabetes and tuberculosis; (5) presence of severe heart, liver, kidney, or coagulation disorders; and (6) pregnant or lactating women or patients with mental illnesses who were unable to cooperate. This study was approved by the Ethics Committee of Nanjing Hospital of Traditional Chinese Medicine (KY2024096). Given the retrospective nature of this study, the requirement for informed consent was waived by the approving body.

### RIFER procedure

All patients in our study underwent subarachnoid block anesthesia. The anesthetic agent used was 0.75% bupivacaine hydrochloride. The anesthesia was administered by experienced anesthesiologists following standard protocols, and the patients’ vital signs were continuously monitored throughout the procedure.

#### Exposure of the inner wall of the fistula tract

The inner wall of the fistula tract was fully exposed. If there was an external opening, mosquito forceps were inserted into the fistula tract through the external opening. Under direct vision, the fistula tract was gradually incised using an electric knife (or ultrasonic scalpel) until the top of the fistula tract was reached, fully exposing the inner wall. In the absence of an external opening, the mucosa and internal sphincter were incised along the dentate line to expose the inner wall of the fistula tract within the intersphincteric space. Mosquito forceps were then inserted into the fistula tract, which was incised using an electric knife (or ultrasonic scalpel) until the top of the fistula tract was reached, fully exposing the inner wall.

#### Excision of the fistula tract

After the inner wall was fully exposed, the fistula tract was completely excised along its outer wall using an electric knife (or an ultrasonic scalpel).

#### Reconstruction of the internal sphincter

After the excision of the fistula tract, the surgical wound was irrigated with diluted povidone-iodine solution to ensure that the wound was clean and to achieve hemostasis. Absorbable sutures (2–0 or 3–0) were then used to suture the broken ends of the internal sphincter on both sides, thereby reconstructing the internal sphincter.

#### Reconstruction of the rectal wall

Absorbable sutures (2–0 or 3–0) were used to perform full-thickness suturing of the intestinal wall from top to bottom until the dentate line, thus completing the reconstruction of the rectal wall.

After the RIFER procedure, patients were transferred to the post-anesthesia care unit for initial monitoring. They were advised to maintain a semi-recumbent position to facilitate wound drainage. Wound care involved regular dressing changes with sterile gauze soaked in diluted povidone-iodine solution. Patients were also provided with dietary advice to ensure a high-fiber diet to prevent constipation, which could put additional stress on the surgical site.

### Data collection and outcome definition

Data on patient demographics and clinical characteristics were collected from electronic medical records. The data included sex, age, height, weight, body mass index (BMI), preoperative assessment tools, and disease duration. The primary evaluation index was the cure rate. Secondary evaluation indices included recurrence rate, wound healing time, occurrence of postoperative complications, visual analog score (VAS), Wexner anal function assessment, incision scar score, appearance of the anus (scar hyperplasia and keyhole-like deformities), and length of hospital stay.

### Statistical analyses

Categorical variables are described using frequencies and percentages. For quantitative data, depending on the distribution, values are presented as mean and standard deviation (normally distributed data) or as median with interquartile range (non-normally distributed data). All statistical analyses were performed using SPSS Statistics version 26 (IBM SPSS Statistics for Windows, Version 26.0; Armonk, NY: IBM Corp).

## Results

### Patient characteristics

This study included 26 patients with intersphincteric anal fistulas who underwent RIFER. The average age of the patients was 36.65 ± 8.68 years (24 male, 92.31%). The baseline patient characteristics are shown in Table 1. The average BMI of the patients was 26.18 ± 3.34 kg/m^2^, and the average disease duration was 352.96 ± 766.59 days (range, 7–3,600 days). All patients underwent an ultrasound evaluation before the operation, and four patients underwent an additional preoperative MRI evaluation. Among the 26 patients with high intersphincteric anal fistulas, eight were found to have branches, as detected by preoperative MRI and endoluminal B-ultrasound examination.

**Table 1:** Characteristics of included patients with high intersphincteric anal fistulas.

Variable	Category	Count or mean	Percentage or SD
Age (years)	-	36.65	8.68
Sex, *n* (%)	Female	2	7.69%
	Male	24	92.31%
Height (cm)	-	173.15	5.54
Weight (kg)	-	78.72	12.25
BMI (kg/m^2^)	-	26.18	3.34
Preoperative assessment tools	B-ultrasound	26	100.0%
	MRI	4	15.38%
Disease duration (days)	-	352.96	766.59

### Therapeutic effects of the RIFER procedure

Among the 26 patients, one had only in-hospital data and was lost to follow-up after discharge. During the 6-month follow-up period, the remaining 25 patients were cured, with no recurrence or postoperative complications (Table 2). The average wound-healing time was 45.40 ± 16.24 days. Throughout the 6-month follow-up period, no cases of wound dehiscence were reported among the 25 patients who completed the follow-up. Surgical wounds were carefully monitored, and the postoperative care protocol was strictly implemented to ensure optimal wound healing.

**Table 2:** Characteristics of pre- and post-operative anal fistula.

Variable	Category	Sample size	Count or mean	Percentage or SD
Outcome of surgery	Cured	25	25	100.00%
	Recurrence	25	0	0
	Wound healing time (days)	25	45.40	16.24
	Postoperative complications	25	0	0
VAS	First defecation	26	1	(1, 2)
	7 days after surgery	26	1	(1, 2)
	14 days after surgery	26	1	(1)
WEXNER	Preoperative	25	0	(0)
	3 months after surgery	25	0	(0, 3)
	6 months after surgery	25	0	(0, 3)
	Incision Scar Score	25	0	(0, 5)
	Appearance of the anus	25	0	0
Length of hospital stay (days)	-	26	10.15	2.94

At the first postoperative defecation, 12 patients had a VAS score of 2, whereas 14 had a score of 1. Seven days postoperatively, three patients had a VAS score of 2, and 23 had a score of 1. Fourteen days postoperatively, all the patients had a VAS score of 1. Preoperatively, all patients had a Wexner anal function score of 0. Three months postoperatively, one patient had a Wexner score of 3, four had a score of 1, and 20 had a score of 0. Six months postoperatively, one patient had a Wexner score of 3, three had a score of 1, and 21 had a score of 0. Three months postoperatively, seven patients had an incision scar score between 1 and 5, while 18 had a score of 0. None of the patients had keyhole-like anal deformities. The mean hospital stay was 10.15 ± 2.94 days.

## Discussion

High anal fistulas are characterized by intricate pathophysiological mechanisms, a risk of incontinence, and a relatively high recurrence rate. The success of high perianal fistula surgery largely depends on the skill and experience of the surgeon and the complexity of the fistula (21). This study used the RIFER procedure, which aimed to preserve anal function by reconstructing the muscles without suturing the fat and skin and preventing infection by leaving the wound semi-open. The results showed a surgical cure rate of 100%, with no recurrence or postoperative complications during the follow-up period. Furthermore, functional outcomes improved, and most patients (72%) had an incision scar score of 0, with no cases of keyhole-like anal deformities. The length of hospital stay, with a mean of 10.15 days in our study, was within the acceptable limits of 7–14 days, as reported in previous studies (22) for similar high intersphincteric anal fistula surgeries. This range allows for proper postoperative monitoring and initial recovery. Similarly, the average wound-healing time of 45.40 days was within the acceptable range of 30–60 days, as per prior research (22), indicating a normal rate of wound healing for this type of surgical procedure.

Previous studies have explored the application of reconstruction in anal fistula surgery. One study evaluated the impact of one-stage complex anal fistula excision with reconstruction of the anal sphincter without stool diversion on fecal incontinence and recurrence. A prospective cohort study of 175 patients with complex high-level perianal fistulas followed up for 1 year after surgery reported that four patients experienced varying degrees of fecal incontinence, 16 experienced recurrence, and five had delayed wound healing (23). Another systematic review included 21 studies that assessed the outcomes of fistulotomy or fistulectomy and immediate sphincter repair (FISR) in terms of healing, incontinence, and sphincter dehiscence, both in general anal fistula cases and high anal fistula cases. The overall healing rate was 93%; however, some patients experienced incontinence and sphincter dehiscence. The healing rate in patients with high anal fistulas was 89%. Although FISR is a safe and effective procedure, inconsistencies in reporting incontinence and defining fistula height, along with limited data on high anal fistulas and significant heterogeneity, make the treatment outcomes for high anal fistulas remain uncertain (24). Innovative surgical techniques combined with reconstruction are necessary to effectively treat high anal fistulas.

The high cure rate of the RIFER procedure is closely related to its unique surgical method. The muscles are reconstructed without suturing the fat and skin, and a semi-open form is adopted to allow better wound drainage and reduce the accumulation of purulent secretions and other substances. Timely discharge of secretions effectively reduces the risk of infection, which promotes wound healing and increases the cure rate (25). Non-suturing of the fat and skin prevents increased local tension and poor blood circulation. Good blood supply and nutrition to the tissues provide favorable conditions for the reconstruction and repair of muscles, which further promotes the success of the surgery and prevents recurrence caused by poor tissue repair.

The RIFER procedure was designed to protect anal function, which is also an important reason for its good therapeutic outcomes. When treating high intersphincteric anal fistulas, this operation precisely reconstructs the muscles, which minimizes damage to key structures, such as the anal sphincter, and maintains the physiological function of the anus. Retention of anal function allows patients to better control their defecation after the operation, preventing fecal incontinence due to sphincter dysfunction (26). Normal anal function maintains local cleanliness and a stable physiological environment, which are beneficial for postoperative wound healing. This reduces the risk of infection and recurrence caused by abnormal anal function, enabling the procedure to achieve a high cure rate while lowering the recurrence rate and incidence of complications (27).

Compared with traditional cutting and non-cutting seton techniques, the RIFER procedure requires a more precise assessment to understand the course, branches of the fistula, and its relationship with the surrounding tissues. A previous study on the cutting-seton technique reported that 70% of the patients were completely cured, 26% had minor complications, 8% of the operated patients experienced mild incontinence, and the recurrence rate was 2% (28). In contrast, in our RIFER procedure study, the complete cure rate was 100%, and both complication and recurrence rates were 0% during the 6-month follow-up period. In terms of preoperative intestinal preparation, traditional surgical methods often utilize conventional measures, such as cleansing enemas. The RIFER procedure may be used to formulate a more personalized intestinal preparation plan based on surgical characteristics, reducing the possibility of intraoperative contamination and postoperative infection, which facilitates smooth surgery and good prognosis.

Although our study showed that the RIFER procedure has good therapeutic effects, there are certain limitations to this study. First, this retrospective study was affected by selection and recall biases. Second, this single-arm study lacked a parallel control group, which may have led to an insufficient objective and comprehensive assessment of the results. Third, the small sample size restricted the possibility of conducting further exploratory analyses, thereby limiting the generalizability of the research conclusions. Fourth, the study did not distinguish between newly diagnosed and recurrent cases, which may have had a significant impact on the treatment effect and interfered with determining the true efficacy of the RIFER procedure. Future studies should increase the sample size and include control groups with different types of anal fistula surgery. Surgical outcomes and associated factors can be analyzed more comprehensively to provide a more reliable basis for clinical treatment.

## Conclusion

The RIFER procedure demonstrated stable and favorable therapeutic outcomes, with a good safety profile for the treatment of patients with high intersphincteric anal fistulas. The cure rate in our study was 100%, and there were no recurrence or postoperative complications. Therefore, the RIFER procedure should be the preferred surgical option in clinical practice for patients with high sphincteric anal fistulas. In light of the limitations of this study, especially the lack of a comparison with the cutting-seton technique, we plan to conduct a prospective, randomized controlled trial in the future. We will recruit a sufficient number of patients with high intersphincteric anal fistulas and randomly assign them to either the RIFER or cutting-seton group. This study will comprehensively evaluate and compare the two techniques in terms of cure rate, recurrence rate, postoperative complications, and long-term anal function, providing more reliable evidence for clinical decision-making.

## Data Availability

The raw data supporting the conclusions of this article will be made available by the authors, without undue reservation.
